# Neuroprotective Effects of Cuscutae Semen in a Mouse Model of Parkinson's Disease

**DOI:** 10.1155/2014/150153

**Published:** 2014-07-22

**Authors:** Minsook Ye, Seul gi Lee, Eun Sook Chung, Su-jin Lim, Won Seob Kim, Heera Yoon, Sun Kwang Kim, Kwang Sung Ahn, Young Pyo Jang, Hyunsu Bae

**Affiliations:** ^1^Department of Physiology, College of Korean Medicine, Kyung Hee University, No. 1, Hoegi-dong, Dongdaemungu, Seoul 130-701, Republic of Korea; ^2^Department of Life and Nanopharmaceutical Sciences, College of Pharmacy, Kyung Hee University, No. 1, Hoegi-dong, Dongdaemungu, Seoul 130-701, Republic of Korea

## Abstract

Parkinson's disease (PD) is a neurodegenerative movement disorder that is characterized by the progressive degeneration of the dopaminergic (DA) pathway. 1-Methyl-4-phenyl-1,2,3,6-tetrahydropyridine (MPTP) causes damage to the DA neurons, and 1-4-methyl-4-phenylpyridinium (MPP^+^) causes cell death in differentiated PC12 cells that is similar to the degeneration that occurs in PD. Moreover, MPTP treatment increases the activity of the brain's immune cells, reactive oxygen species- (ROS-) generating processes, and glutathione peroxidase. We recently reported that Cuscutae Semen (CS), a widely used traditional herbal medicine, increases cell viability in a yeast model of PD. In the present study, we examined the inhibitory effect of CS on the neurotoxicity of MPTP in mice and on the MPP+-induced cell death in differentiated PC12 cells. The MPTP-induced loss of nigral DA neurons was partly inhibited by CS-mediated decreases in ROS generation. The activation of microglia was slightly inhibited by CS, although this effect did not reach statistical significance. Furthermore, CS may reduce the MPP+ toxicity in PC12 cells by suppressing glutathione peroxidase activation. These results suggest that CS may be beneficial for the treatment of neurodegenerative diseases such as PD.

## 1. Introduction

Parkinson's disease (PD) is a progressive neurodegenerative disorder that is characterized by resting tremor, rigidity, slowed movements, and bradykinesia, which result from the loss of substantia nigra (SN) dopaminergic (DA) neurons and their projections to the striatum (STR) [1–4]. The etiology of this disease is unknown, but genetic, environmental, age-related, and inflammatory processes are factors that affect disease onset and progression [5–8]. The pathological hallmark of PD is the loss of DA neurons in the SN that project to the STR, which plays a fundamental role in normal motor function [4]. Clinical parkinsonian symptoms occur when 60 to 70 percent of SN DA neurons are lost, and losses in this range result in decreases in dopamine levels in the nigrostriatal system [9–11]. Although PD is a sporadic disease of unknown pathogenesis, accumulating evidence suggests that glial activation-derived oxidative stress increases the risk of developing PD [12].* In vivo* and* in vitro* 1-methyl-4-phenyl-1,2,3,6-tetrahydropyridine (MPTP) models of PD have shown that key enzymes involved in the production of reactive oxygen species (ROS) are upregulated in damaged areas and contribute to the death of DA neurons [13–16].

Cuscutae Semen (CS) has been widely used in traditional herbal medicine to modulate immune-related diseases such as hepatotoxicity and osteoblast differentiation [17, 18]. Several studies have suggested that CS invigorates the reproductive system and improves kidney deficits in animal models [19, 20]. Recent research has shown that CS affects oxidative stress in the brains of aged rodents [1, 2]. Additionally, CS has been shown to enhance lymphocyte proliferation both* in vitro* and* in vivo* [21, 22]. These reports suggest that CS has the potential to modulate immune responses. Although a number of the components of CS have been identified, the mechanisms of and ingredients responsible for the immunomodulatory effects of CS remain to be illuminated.

In the present study, we sought to determine whether CS promotes the survival of DA neurons both in neuronal differentiated PC12 cells and the MPTP-induced mouse model of PD. We also investigated whether CS was ultimately associated with reductions in ROS generation and oxidative stress.

## 2. Materials and Methods

### 2.1. Reagents

Water-extracted dried Cuscutae Semen (CS) was purchased from Sun Ten Pharmaceutical (Taipei, Taiwan). Water extraction of Cuscutae Semen was performed following the manufacturer's protocol. First, the Cuscutae Semen was water-extracted at 100°C for 60 min. Then, the remains of the herbs and impurities were separated from the extracted liquid in a filtration separation process. The water extracts were then spray dried, and corn starch was added as an excipient to stabilize the concentrated herbal products (final ratio of water extracts versus starch of 7 : 3). After these procedures, the final product of Cuscutae Semen extract (ST) was produced in Sun Ten. The Cuscutae Semen extract powder was dissolved in PBS. MPTP-HCl (20 mg/kg, free base in saline; Sigma-Aldrich, St. Louis, MO) was also dissolved in PBS.

**Figure 1:**
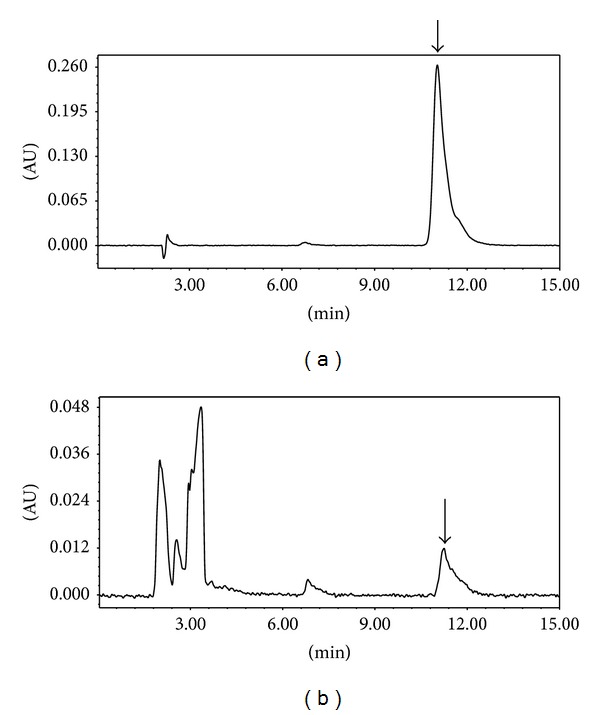
The HPLC analysis of the standard material to CS. Phytochemical analysis was performed by HPLC as described in the materials and methods section. Kaempferol was utilized as an authentic standard (arrows) and it was found that the concentration of kaempferol in CS was calculated to be 0.24 ± 0.01 *μ*g/g. (a) HPLC chromatogram of kaempferol standard solution, (b) methanol extract of Cuscutae Semen granule.

For the phytochemical analysis of CS, high-performance liquid chromatography (HPLC) was performed. The CS was accurately weighed to 500 mg and then dissolved in 50 mL 100% methanol. The sample was extracted in an ultrasonic bath for 60 min at 50°C. The suspension was filtered, and the filtrate was evaporated in vacuo. The residue was dissolved in methanol (500 *μ*L), and the obtained solution was filtered through a membrane filter (0.45 *μ*m pore size) prior to injection. For the quantitative analysis of CS, kaempferol, one of the known flavonol constituents of CS, was diluted in acetonitrile to five different concentrations (25, 50, 100, 200, and 500 ppm). Each solution was filtered through a membrane filter (0.45 *μ*m pore size) prior to injection. The HPLC analysis was performed using a Waters Alliance system equipped with an in-line degasser and a photodiode array detector. The UV spectra were collected across the range of 200 to 500 nm and monitored at 360 nm for chromatograms. Empower software was used for instrument control, data collection, and data processing. The HPLC column was an Inspire C18 (4.6 × 250 mm, 5 *μ*m). The mobile phase was an isocratic system with acetonitrile (A) phosphoric acid 0.25% (B) run for 15 min. The ratio of A : B was 39 : 61. The flow rate was 1 mL/min. The injection volume for all samples and standard solutions was 10 *μ*L. Using this method, the concentration of kaempferol in CS was calculated to be 0.24 ± 0.01 *μ*g/g as described in Figure 1.

### 2.2. Cell Culture and Drug Treatment

PC12 cells were obtained from the Korean Cell Line Bank. PC12 cells were cultured in Dulbecco's modified Eagle's media (DMEM, Welgene) supplemented with 10% fetal bovine serum (FBS, Welgene), 5% horse serum (HS, Welgene), 1% penicillin, and 1% streptomycin in a water-saturated atmosphere of 5% CO_2_ at 37°C. PC12 cells were incubated in 50 ng/mL nerve growth factor (NGF) for differentiation. MPP+ (Sigma-Aldrich, St. Louis, MO) and CS (Sun Ten Pharmaceutical, Taipei, Taiwan) were dissolved in PBS and prepared as a 10 mmol/L stock immediately before use. The drugs were diluted in culture medium to the indicated final concentrations. The total volume of the reaction system in the following experiments was 5 mL.

### 2.3. Measurement of Cell Viability

Intracellular ROS was evaluated using the fluorescent probe 2,7-dichlorofluorescein diacetate (H_2_CDFDA). H_2_CDFDA is oxidized to the highly fluorescent compound dichlorofluorescein (DCF) by intracellular hydroperoxides. The generation of DCF is proportional to intracellular ROS levels. After exposure to 75 *μ*mol/L MPP+ in the presence or absence of CS for 24 h, PC12 cells were incubated with 10 *μ*mol/L H_2_CDFDA (Sigma-Aldrich, St. Louis, MO) at 37°C for 1 h. The cells were washed twice with FBS-free media. Fluorescence was measured at an excitation wavelength of 485 nm and an emission wavelength of 538 nm using a fluorescence microplate reader.

### 2.4. Measurement of Intracellular Reactive Oxygen Species Formation

Intracellular ROS was evaluated using the fluorescent probe 2, 7-dichlorofluorescein diacetate (H_2_CDFDA). H_2_CDFDA is oxidized to the highly fluorescent compound dichlorofluorescein (DCF) by intracellular hydroperoxides. The generation of DCF is proportional to intracellular ROS levels. After exposure to 75 *μ*mol/L MPP+ in the presence or absence of CS for 24 h, PC12 cells were incubated with 10 *μ*mol/L H_2_CDFDA (Sigma-Aldrich, St. Louis, MO) at 37°C for 1 h. The cells were washed twice with FBS free media. Fluorescence was measured at an excitation wavelength of 485 nm and an emission wavelength of 538 nm using a fluorescence microplate reader.

### 2.5. Measurement of Glutathione Peroxidase Activity

The effect of CS treatment on GSH level was measured using a commercial kit (Cayman Chemical Co., Ann Arbor, MI, USA). The homogenate of PC12 cells was used for GSH measurement with the GSH Assay Kit (Cayman Chemical Co., Ann Arbor, MI, USA) following the manufacturer's protocol. The GSH content was determined by comparison with the standards and normalized to protein content.

### 2.6. Animals and MPTP Administration

All of the experiments were conducted with 7- to 8-week-old male C57BL/6J mice (weighing 21-22 g; Japan SLC Inc., Hamamatsu, Japan). The mice were maintained in a room at 20–22°C on a 12 h light/dark cycle with food supplied ad libitum. The study was approved by the University of Kyung Hee Animal Care and Use Committee. Mice were randomly divided into five groups of six mice each. For MPTP intoxication, the mice received four intraperitoneal (i.p.) injections of MPTP-HCl (20 mg/kg, free base in saline; Sigma-Aldrich, St. Louis, MO) dissolved in PBS at 2 h intervals. Twelve hours after the last MPTP injection, the MPTP-injected mice received single daily p.o. administrations of CS (50, 100, or 200 mg/kg) or PBS for 6 days. Control mice were administered either CS or vehicle alone.

### 2.7. Measurement of Motor Activity

The pole test has been utilized to measure motor coordination and balance in mouse models of PD [3]. We performed the pole test on the 7th day after the last MPTP injection. In this test, animals were placed on top of a rough-surfaced iron pole (50 cm in length and 0.8 cm in diameter) and allowed to climb down to the base of the pole. The times that it took for the mouse to turn completely downward (time to turn; T-turn) and then to reach the floor (locomotion activity time; T-LA) were recorded, with a cut-off limit of 30 s. The average of the best three measurements was used as the result.

### 2.8. Tissue Preparation and Immunostaining

The mice were transcardially perfused with a saline solution containing 0.5% sodium nitrate and heparin (10 U/mL) and then fixed with 4% paraformaldehyde (PFA) dissolved in 0.1 M phosphate buffer (PB). The brains were dissected from the skulls, postfixed overnight in buffered 4% paraformaldehyde at 4°C, stored in a 30% sucrose solution at 4°C until they sank, and frozen sectioned on a sliding microtome in 30 *μ*m thick coronal sections. All sections were collected in six separate series and processed for immunostaining as described previously [20]. In brief, the brain sections were rinsed in PBS and then incubated overnight at room temperature with the primary antibodies. The following day, the brain sections were rinsed with PBS and 0.5% bovine serum albumin (BSA), incubated with the appropriate biotinylated secondary antibody, and processed with an avidin-biotin complex kit (Vectastain ABC kit; Vector Laboratories, Burlingame, CA). The bound antiserum was visualized by incubation with 0.05% diaminobenzidine-HCl (DAB) and 0.003% hydrogen peroxide in 0.1 M PB. The DAB reaction was stopped by rinsing the tissues in 0.1 M PB. The primary antibodies included antibodies directed against tyrosine hydroxylase (TH; 1 : 2000, Pel-freez, Brown Deer, WI) and CD11b (1 : 200; Serotec, Oxford, UK). Labeled tissue sections were then mounted on gelatin-coated slides and analyzed under a bright-field microscope (Nikon, Tokyo, Japan).

### 2.9. Stereological Cell Counting

Unbiased stereological estimations of the total numbers of TH-positive DA neurons and CD11b-positive microglia/macrophages in the SN were made using the optical fractionator method, which was performed on an Olympus computer assisted stereological toolbox (CAST) system (version 2.1.4, Olympus, Ballerup, Denmark) as previously described [21]. The sections used for counting covered the entire SN from the rostral tip of the pars compacta to the caudal end of the pars reticulata (anterioposterior: −2.06 to −4.16 mm from bregma). The actual counting was performed using a 1003 oil objective. The total number of cells was estimated according to the optical fractionator equation [22]. More than 300 points across all sections from each specimen were analyzed.

### 2.10. Densitometry Analysis

As previously described [5], an average of 17 coronal sections of the STR, starting rostrally from +1.60 mm anteroposterior and extending caudally to 0.00 mm, were examined at a 5x magnification using the IMAGE PRO PLUS system (version 4.0; Media Cybernetics, Silver Spring, MD) on a computer attached to a light microscope (Zeiss Axioskop, Oberkochen, Germany) and interfaced with a video camera with a charge-coupled device (Kodak Mega Plus model 1.4 I). To determine the density of the TH-immunoreactive staining in the STR, a square frame of 700 × 700 *μ*m was placed in the dorsal part of the STR. A second square frame of 200 × 200 *μ*m was placed in the region of the corpus callosum to measure background values. To control for variations in background illumination, the average of the background density readings from the corpus callosum was subtracted from the average of the density readings of the STR for each section as described previously [5]. Next, the average of all sections was calculated individually for each animal before the data were statistically processed.

### 2.11. In Situ Detection of O_2_^−^- and O_2_^−^-Derived Oxidants

Three days after final MPTP injection, hydroethidine (Molecular Probes; 1 mg/mL in PBS containing 1% dimethyl sulfoxide) was administered intraperitoneally. After 15 min, the animals were transcardially perfused and postfixed, and the brains were cut into 30 *μ*m sections. Hydroethidine histochemistry was performed for the in situ visualization of O_2_^−^- and O_2_^−^-derived oxidants as previously described [23]. The oxidized hydroethidine product, ethidium, was examined by confocal microscopy (Olympus).

### 2.12. Statistical Analyses

All values are expressed as the means ± the SEMs. The statistical significance (*P* < 0.05 for all analyses) of comparisons across multiple groups was assessed with one-way or two-way ANOVAs, and Student's *t*-tests were used for single comparisons. All statistical analyses were performed with Prism 5.01 software (GraphPad Software Inc., San Diego, CA, USA).

## 3. Results

### 3.1. Effects of CS on ROS Formation and GSH Depletion in MPP+-Induced PC12 Cells

**Figure 2:**
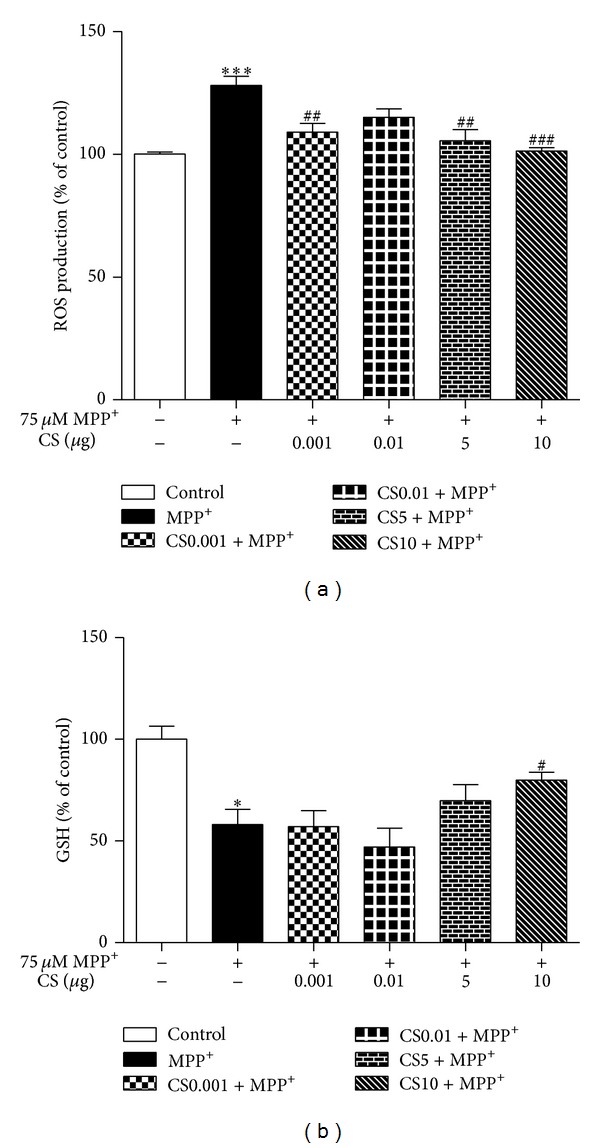
Effects of CS on the formation of reactive oxygen species (ROS) and glutathione (GSH) depletion due to MPP+. The PC12 cells (1 × 10^5^ cells/mL) were treated with 1 mM MPP+ in the presence of CS (0.001, 0.01, 5, and 10 *μ*g) for 24 h at 37°C. Formation of ROS was assayed by measuring fluorescence of dichlorofluorescein (DCF, a); GSH contents (*μ*mol/mg protein) were determined using GSH reductase (b). Data are expressed as the mean ± S.E.M. *P < 0.05, ***P < 0.001 compared with control. ^#^ *P* < 0.05, ^##^ *P* < 0.01, ^###^ *P* < 0.001 compared with MPP^+^.

After exposure to 75 *μ*mol/L MPP+, the fluorescence intensity of DCF increased to about twice the control value. CS prevented the MPP+-induced increase in DCF fluorescence and showed a maximal inhibitory effect at 10 *μ*g (Figure 2). ROS formation was not further attenuated by 0.01 *μ*g concentrations of CS.

A reduction in GSH levels increases the sensitivity of neurons to the toxic effect of neurotoxins [24] and is associated with mitochondrial dysfunction [25]. In the present study, we assessed the effect of CS on the MPP+-induced decrease in GSH. CS prevented the MPP+-induced decrease in GSH (Figure 2).

### 3.2. Inhibitory Effects of CS on Behavioral Impairment by MPTP in Mice

**Figure 3:**
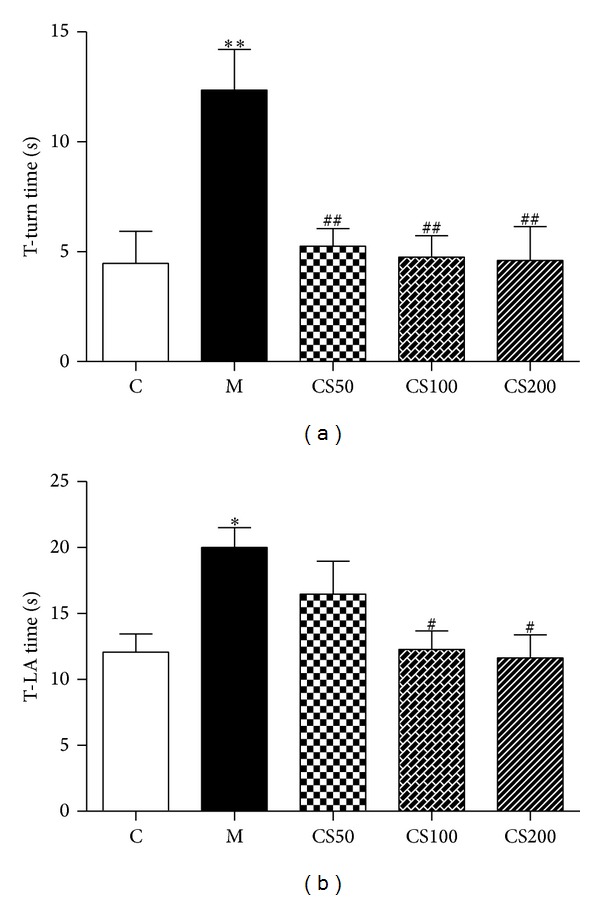
Effect of CS on MPTP-induced behavioral deficits in mice. Mice were pretreated with CS (50, 100, or 200 mg/kg) for six days and then injected with MPTP (20 mg/kg i.p.). The time taken for the mice to turn completely downward ((a); T-turn) and the time taken to reach the floor ((b); T-LA) were recorded. Values are indicated as the mean ± SEM. *P < 0.05, **P < 0.01 compared with the control group, ^#^ *P* < 0.05, ^##^ *P* < 0.01 compared with the MPTP-only treated group.

To confirm the effect of AJW on dopaminergic neurons in an* in vivo* PD model, we treated mice with CS and/or MPTP and carried out the pole test. As a result, the T-turn and T-LA of MPTP-only treated mice were markedly prolonged at 12.35 ± 1.85 sec and 20.02 ± 1.49 sec compared with the control group (T-turn: 4.47 ± 1.46 sec; T-LA:  12.07 ± 1.38 sec) (Figure 3). However, the times of the CS 50 mg/kg/day treated group were shortened to 16.47 ± 2.5 sec for the T-turn, and the CS 200 mg/kg/day treated group showed a more shortened T-turn at 4.61 ± 1.53 sec and T-LA at 11.64 ± 1.74 sec (Figure 3).

### 3.3. CS Protects DA Neurons in the SN against MPTP Neurotoxicity

**Figure 4:**
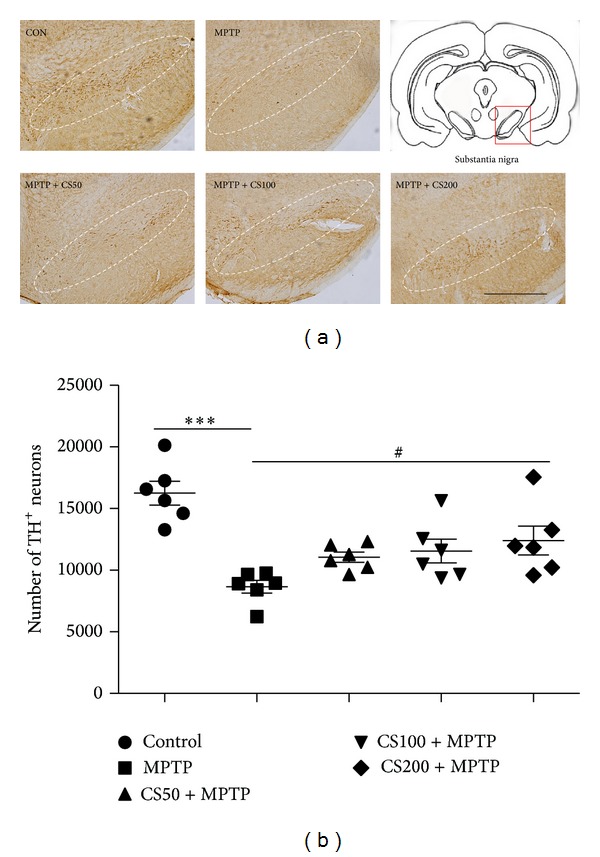
CS protects against DA neuronal death due to MPTP-induced neurotoxicity. At 7 days after the final MPTP injection, the animals were sacrificed, and the brains were prepared for immunohistochemistry. Tyrosine hydroxylase (TH) immunostaining of the SNs of the mice treated with PBS (control), MPTP, and vehicle or MPTP and CS (50, 100, or 200 mg/kg). The representative images were displayed (a). The scale bars represent 500 *μ*m. The number of TH-positive neurons in the SN measured (b). At 7 days after the final MPTP injection, the animals were sacrificed, and the brains were prepared for immunohistochemistry. The error bars represent the SEMs. ^#^ *P* < 0.05, significantly different from the MPTP/PBS treated control group. ***P < 0.001, significantly different from the saline with PBS group (one-way ANOVA and Student-Newman-Keuls analyses). Dotted lines indicate the SN. The schematic brain sections are from the atlas (Paxinos and Watson, 1986). The square represents SN in the mouse brain. C: control; M: MPTP; CS50: CS50+MPTP; CS100: CS100+MPTP; CS200: CS200+MPTP.

In the brain, MPTP is metabolized to 1-methyl-4-phenylpyridinium (MPP+), which is the active toxic compound that is primarily responsible for MPTP-induced neurotoxicity [24]. MPTP was rapidly eliminated and nearly undetectable at 6 h, and striatal MPP+ levels peaked within 30 min and subsequently declined steadily until levels no longer differed significantly from the control after 12 h [21]. Accordingly, CS was administered 12 h after the last injection of MPTP for all* in vivo* experiments to avoid interference with the metabolism of MPTP. To examine the neuroprotective effects of CS, the MPTP-injected mice were treated with CS (50, 100, or 200 mg/kg) or PBS for 6 days. Seven days after the MPTP injections, brain sections were immunostained with an anti-TH antibody to detect DA neurons (Figure 4(a)). In MPTP-injected mice, there was a significant loss of TH^+^ neurons in the SN (56%; *P* < 0.001; Figure 4(b)), and the density of TH^+^ fibers in the STR was reduced by 51% (*P* < 0.001; Figure 4(b)) compared to the PBS-injected control mice. CS treatment effectively blocked DA neuronal damage in the SN by 17% (*P* < 0.05; Figure 2(b)) compared to the PBS-injected control mice.

### 3.4. CS Protects DA Fibers in the STR against MPTP Neurotoxicity

**Figure 5:**
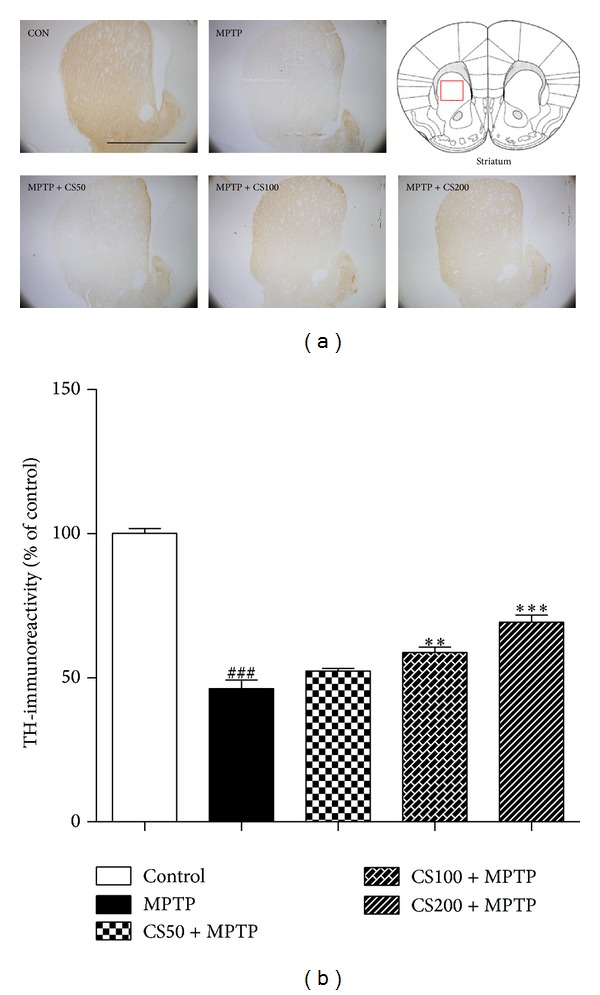
*In vivo* MPTP-induced DA fiber neurotoxicity in the STR. The treatment of the MPTP-injected mice with CS prevented DA neuronal death in the STR. MPTP-injected mice were treated with CS (50, 100, or 200 mg/kg) or PBS for 6 days beginning 12 h after the final MPTP injection. Seven days after the MPTP injections, brain sections were prepared and immunostained with TH antibody to identify DA fibers. The representative images were displayed (a). Scale bar was 5 mm. The densities of the TH-fibers in the STR were stereologically counted (b). The error bars represent the SEMs. **P < 0.01, ***P < 0.001, significantly different from the MPTP with PBS group. ^###^ *P* < 0.001, significantly different from the saline with PBS group (one-way ANOVA and Student-Newman-Keuls analyses). The schematic brain sections are from the atlas (Paxinos and Franklin, 1997). The square represents STR in the mouse brain. C: control; M: MPTP; CS50: CS50+MPTP; CS100: CS100+MPTP; CS200: CS200+MPTP.

In the brain, MPTP is metabolized to 1-methyl-4-phenylpyridinium (MPP+), which is the active toxic compound that is primarily responsible for the neurotoxicity of MPTP [4]. MPTP was rapidly eliminated and nearly undetectable at 6 h, and striatal MPP+ levels peaked within 30 min and subsequently declined steadily until they were no longer significantly different from the controls after 12 h [5]. Accordingly, CS was administered 12 h after the final injection of MPTP in all* in vivo* experiments to avoid interfering with the metabolism of MPTP. To examine the neuroprotective effect of CS, MPTP-injected mice were treated with CS (50, 100, or 200 mg/kg) or PBS for 6 days. Seven days after the MPTP injection, brain sections were immunostained with an anti-TH antibody to detect DA neurons (Figure 5(a)). In the MPTP-injected mice, there was a significant reduction in the density of TH^+^ fibers in the STR (55%; *P* < 0.001; Figure 5(b)) compared to the PBS-injected control mice. CS treatment effectively blocked the reduction in the density of TH^+^ fibers in the STR by 23% (*P* < 0.001; Figure 5(b)) compared to the MPTP mice.

### 3.5. Effectiveness of CS against Microglia-Derived Neurotoxicity

**Figure 6:**
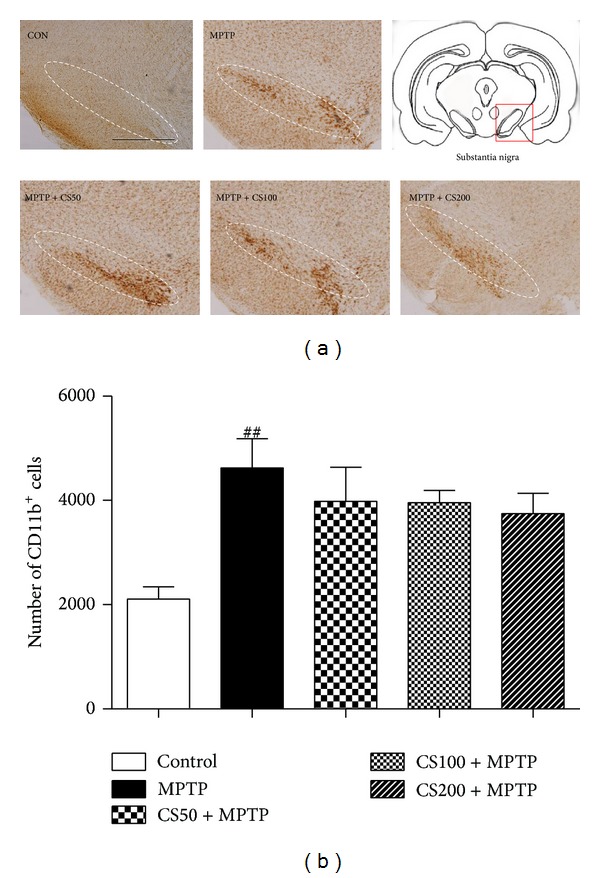
MPTP-induced microglial activation is inhibited by CS. Seven days after the final MPTP injection, the animals were sacrificed, and the brains were prepared for immunohistochemistry. CD11b immunostainings in the SN of mice treated with PBS (control), MPTP, and vehicle or MPTP and CS (50, 100, or 200 mg/kg) are shown. The representative images were displayed (a). The scale bars represent 500 *μ*m. The numbers of CD11b-positive cells in the SN were stereologically counted (b). The error bars represent the SEMs. ^##^ *P* < 0.001, significantly different from saline with PBS (one-way ANOVA and Student-Newman-Keuls analyses). Dotted lines indicate the SN. The schematic brain sections are from the atlas (Paxinos and Watson, 1986). The square represents SN in the mouse brain. C: control; M: MPTP; CS50: CS50+MPTP; CS100: CS100+MPTP; CS200: CS200+MPTP.

Activated microglia play important roles in the death of DA neurons in PD patients [6, 7] and the MPTP-induced animal model of PD [5]. To determine whether the neuroprotective effect of CS resulted from the inhibition of microglial activation in the SN, we performed immunostaining with anti-CD11b to detect microglia/macrophages in brain sections that were prepared 7 days after the final MPTP injection (Figure 6). In saline-treated control mice, few CD11b^+^ microglia/macrophages with resting morphologies (i.e., small cell bodies and thin processes) were observed in the SN on the 7th day. In contrast, numerous CD11b^+^ microglia/macrophages with activated morphologies (i.e., larger cell bodies and thick processes) were apparent in the SN of the MPTP-injected mice on the 7th day. In the MPTP-injected mice treated with CS, activated microglia/macrophages also appeared in the SN on the 7th day after MPTP injection. Thus, CS had no effect on CD11b^+^ microglia/macrophage activation in the SN.

### 3.6. CS Reduces Oxidant Production in the SN of MPTP-Injected Mice

**Figure 7:**
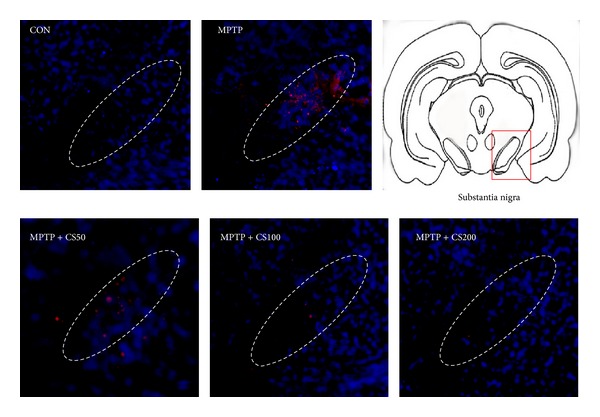
MPTP-induced ROS generation is inhibited by CS. CS inhibits MPTP-induced O_2_^−^ and O_2_^−^-derived oxidant production and protein oxidation in the SN. The animals received hydroethidine 48 h after the intranigral injection of MPTP in the absence or presence of CS. After 45 min, the brains were harvested, and sections of the SN were prepared for hydroethidine histochemistry to detect extracellular superoxide. Confocal microscopic observation revealed ethidium fluorescence (red) in the SN. The nuclei were counterstained with Hoechst 33258 (blue). These data are representative of five to six animals per group. Dotted lines indicate the SN. The schematic brain sections are from the atlas (Paxinos and Watson, 1986). The square represents SN in the mouse brain.

Recent studies have suggested that activated microglia produce O_2_^−^- and O_2_^−^-derived oxidants [8, 12]. We investigated whether CS enhanced DA neuron survival by inhibiting MPTP-induced ROS production. The fluorescent products of oxidized hydroethidine (i.e., ethidium accumulation) were significantly increased at 48 h in the SNs of the MPTP-injected mice (Figure 7) compared to the PBS-injected controls (Figure 7). This MPTP-induced oxidant production was dramatically decreased by CS (Figure 7).

## 4. Discussion

In this study, the protective effects of CS were verified* in vitro* using a PD cellular model induced by MPP+ in PC12 cells and* in vivo* using a PD model induced by MPTP administration in mice. We showed that CS suppressed MPTP-induced ROS generation and reduced oxidative stress, which led to the survival of nigrostriatal DA neurons. At the cellular level, CS seems to reduce the MPP+-induced cell viability as well as ROS and GSH levels. To our knowledge, this is the first study to show that CS prevents nigrostriatal DA neuronal death via the blockade of ROS generation in the MPTP model of PD.

Previous studies have described the components of CS, such as quercetin, kaempferol, and hyperoside, as well as their various pharmacological activities [19, 26, 27]. In one of these studies, kaempferol was shown to attenuate the immune function of dendritic cells [26]. Some studies have suggested that CS acts on defective kidneys and the reproductive system in animal models [19, 20] and induces neuronal differentiation. Moreover, CS has hepatoprotective and antioxidant effects against acetaminophen-induced hepatotoxicity [18]. CS has also been shown to be neuroprotective in a psychological stress-induced rat due to its actions on the hypothalamus-pituitary-ovary axis [28, 29]. Additionally, CS has been demonstrated to have protective effects in D-galactose-induced mimetic aging mice [1] and aging rats [2] that are mediated by improvements in the antioxidant statuses of these animals. Based on these findings, CS is relevant to PD because of its neuroprotective effects and its suppression of ROS.

The administration of CS had protective effects against the severe reduction in the levels of TH immunoreactivity in the striatum and SN after MPTP treatment. These results seem to suggest that CS can protect against the neuronal damage caused by MPTP toxicity.

Microglia are the resident macrophages of the brain and play crucial roles in the development and preservation of the neural environment [30]. However, in the presence of reverse stimuli, microglia can induce chronic, damaging inflammation that ultimately leads to neuronal cell death. Microglia are the intrinsic immune effector cells that are activated in response to neuronal damage [31]. Several direct neurotoxins can activate microglia through the production of various factors, including ROS and proinflammatory cytokines [8, 32].

The conversion of MPTP to MPP+ by monoamine oxidase-B (MAO-B) in astrocytes is followed by the accumulation of MPP+ in the DA neurons of the SN due to the activity of DAT. This accumulation within the DA neurons results in the generation of reactive oxygen species (ROS) by the mitochondria, including nitric oxide (NO), superoxide anion (O_2_^−^), hydrogen peroxide (H_2_O_2_), and hydroxyl radicals (^•^OH) [33, 34]. ROS may result in oxidative stress to the DA neurons and induce and/or aggravate neurotoxicity. Some studies have implicated oxidative stress as a major cause of neuronal injury in neurological diseases such as PD [35–37]. As a pathogenic condition, oxidative damage may account for the degeneration of DA neurons in the SN of PD brains [38, 39] and in the brains of MPTP-treated mice [40]. The ROS responsible for these molecular modifications can be generated by microglial NADPH oxidase and play important roles in the development of oxidative stress in the MPTP model of PD. These data support the hypothesis that the observed neuroprotective effects of CS were mediated by reductions in oxidative damage.

To evaluate the toxicity of MPTP on cell viability, we used the toxicity of MPP+ instead of MPTP against differentiated PC12 cells. Upon nerve growth factor stimulation, PC12 cells not only reveal abundant neuritic growth but also choose a neurochemical dopaminergic phenotype [41]. MPP+-induced cellular toxicity is mediated through ROS generation [42]. In line with a previous study [43], PC12 cells produced a significant amount of intracellular ROS when exposed to 75 *μ*M MPP+ for 24 h. However, pretreatment with CS suppressed the MPP+-induced accumulation of ROS. These findings show that CS exhibits broad-spectrum antioxidant activity. Oxidative stress results from a redox imbalance between the formation and deletion of ROS. Glutathione (GSH) is the most plentiful antioxidant in cells and plays an important role in cellular defense against oxidative stress [44]. In the present study, MPP+ induced a marked decline in the total GSH level in PC12 cells, whereas pretreatment with CS significantly restored the MPP+-induced decrease in total GSH.

CS has anti-inflammatory activities that reduce the production of cytokines and chemokines by mouse bone marrow-derived dendritic cells when these cells are stimulated with LPS [45]. However, CS was not found to be effective in reducing the microglial activation in the SN of MPTP-injected mice. MPTP directly damages DA neurons when it is taken up through the DA transporter; this uptake results in oxidative mitochondrial damage, which leads to neuronal death. MPP+-induced neuronal damage activates microglia [16] to produce some potentially toxic substances that can damage neurons via an indirect mechanism. Our results imply that the neuroprotective effects of CS are associated with a blockade of oxidative stress in DA neurons and are not associated with the microglial activation that occurs in the MPTP-induced PD animal model.

## 5. Conclusion

In conclusion, CS protected dopaminergic cells from MPP+ toxicities in PC12 cells and against MPTP toxicities in C57BL/6 mice.* In vitro*, the neuroprotective effects of CS are due to its antioxidant activities, including the inhibition of ROS and an increase in GSH.* In vivo*, CS inhibited the production of ROS, prevented oxidative damage to neurons, and led to increased neuronal survival. These results suggest that CS may be beneficial as a novel therapeutic agent for neurodegenerative diseases that are related to oxidative damage, including PD.

## References

[1] Liu JH, Ho S, Lai T, Liu T, Chi P, Wu R (2003). Protective effects of Chinese herbs on D-galactose-induced oxidative damage. *Methods and Findings in Experimental and Clinical Pharmacology*.

[2] Wang XM, Fu H, Liu GX (2001). Effect of wuzi yanzong pill and its disassembled prescription on mitochondrial DNA deletion, respiratory chain complexes and ATP synthesis in aged rats. *Zhongguo Zhong Xi Yi Jie He Za Zhi*.

[3] Ogawa N (1997). Dopamine neurotransmission and treatments for Parkinson's disease in the molecular biology era. *European Neurology*.

[4] Przedborski S, Jackson-Lewis V, Naini AB (2001). The parkinsonian toxin 1-methyl-4-phenyl-1,2,3,6-tetrahydropyridine (MPTP): a technical review of its utility and safety. *Journal of Neurochemistry*.

[5] Chung YC, Kim SR, Park J (2011). Fluoxetine prevents MPTP-induced loss of dopaminergic neurons by inhibiting microglial activation. *Neuropharmacology*.

[6] Hirsch EC, Breidert T, Rousselet E, Hunot S, Hartmann A, Michel PP (2003). The role of glial reaction and inflammation in Parkinson's disease. *Annals of the New York Academy of Sciences*.

[7] McGeer PL, McGeer EG (2008). Glial reactions in Parkinson's disease. *Movement Disorders*.

[8] Block ML, Zecca L, Hong J-S (2007). Microglia-mediated neurotoxicity: uncovering the molecular mechanisms. *Nature Reviews Neuroscience*.

[9] Bernheimer H, Birkmayer W, Hornykiewicz O, Jellinger K, Seitelberger F (1973). Brain dopamine and the syndromes of Parkinson and Huntington Clinical, morphological and neurochemical correlations. *Journal of the Neurological Sciences*.

[10] Riederer P, Wuketich S (1976). Time course of nigrostriatal degeneration in Parkinson's disease. A detailed study of influential factors in human brain amine analysis. *Journal of Neural Transmission - General Section*.

[11] Lang AE, Lozano AM (1998). Parkinson's disease. First of two parts. *The New England Journal of Medicine*.

[12] Koutsilieri E, Scheller C, Tribl F, Riederer P (2002). Degeneration of neuronal cells due to oxidative stress—microglial contribution. *Parkinsonism and Related Disorders*.

[13] Liberatore GT, Jackson-Lewis V, Vukosavic S (1999). Inducible nitric oxide synthase stimulates dopaminergic neurodegeneration in the MPTP model of Parkinson disease. *Nature Medicine*.

[14] Choi D-K, Pennathur S, Perier C (2005). Ablation of the inflammatory enzyme myeloperoxidase mitigates features of Parkinson's disease in mice. *The Journal of Neuroscience*.

[15] Wu D, Teismann P, Tieu K (2003). NADPH oxidase mediates oxidative stress in the 1-methyl-4-phenyl-1,2,3,6-tetrahydropyridine model of Parkinson's disease. *Proceedings of the National Academy of Sciences of the United States of America*.

[16] Gao H, Liu B, Zhang W, Hong J (2003). Critical role of microglial NADPH oxidase-derived free radicals in the in vitro MPTP model of Parkinson’s disease. *The FASEB Journal*.

[17] Yang L, Chen Q, Wang F, Zhang G (2011). Antiosteoporotic compounds from seeds of *Cuscuta chinensis*. *Journal of Ethnopharmacology*.

[18] Yen FL, Wu T, Lin L, Cham T, Lin C (2008). Nanoparticles formulation of Cuscuta chinensis prevents acetaminophen-induced hepatotoxicity in rats. *Food and Chemical Toxicology*.

[19] Qin D, She B, She Y, Wang J (2000). Effects of flavonoids from Semen Cuscutae on the reproductive system in male rats. *Asian Journal of Andrology*.

[20] Yang HM, Shin H, Kang Y, Kim J (2009). Cuscuta chinensis extract promotes osteoblast differentiation and mineralization in human osteoblast-like MG-63 cells. *Journal of Medicinal Food*.

[21] Bao X, Wang Z, Fang J, Li X (2002). Structural features of an immunostimulating and antioxidant acidic polysaccharide from the seeds of Cuscuta chinensis. *Planta Medica*.

[22] Wang Z, Fang J-N, Ge D-L, Li X-Y (2000). Chemical characterization and immunological activities of an acidic polysaccharide isolated from the seeds of *Cuscuta chinensis* Lam. *Acta Pharmacologica Sinica*.

[23] Chung ES, Chung YC, Bok E (2010). Fluoxetine prevents LPS-induced degeneration of nigral dopaminergic neurons by inhibiting microglia-mediated oxidative stress. *Brain Research*.

[24] Zeevalk GD, Bernard LP, Albers DS, Mirochnitchenko O, Nicklas WJ, Sonsalla PK (1997). Energy stress-induced dopamine loss in glutathione peroxidase-overexpressing transgenic mice and in glutathione-depleted mesencephalic cultures. *Journal of Neurochemistry*.

[25] Pereira CF, Oliveira CRD (2000). Oxidative glutamate toxicity involves mitochondrial dysfunction and perturbation of intracellular Ca2^+^ homeostasis. *Neuroscience Research*.

[26] Ye M, Li Y, Yan Y, Liu H, Ji X (2002). Determination of flavonoids in *Semen Cuscutae* by RP-HPLC. *Journal of Pharmaceutical and Biomedical Analysis*.

[27] Wu L, Yang X, Huang Z, Liu H, Wu G (2007). *In vivo* and *in vitro* antiviral activity of hyperoside extracted from *Abelmoschus manihot* (L) medik. *Acta Pharmacologica Sinica*.

[28] Wang J, Wang M, Ou Y, Wu Q (2002). Effects of flavonoids from semen Cuscutae on changes of beta-EP in hypothalamuses and FSH and LH in anterior pituitaries in female rats exposed to psychologic stress. *Journal of Chinese medicinal materials*.

[29] Ke J, Duan R (2013). Effects of flavonoids from semen cuscutae on the hippocampal-hypothalamic- pituitary-ovarian sex hormone receptors in female rats exposed to psychological stress. *Clinical and Experimental Obstetrics and Gynecology*.

[30] Kraft AD, Harry GJ (2011). Features of microglia and neuroinflammation relevant to environmental exposure and neurotoxicity. *International Journal of Environmental Research and Public Health*.

[31] Block ML, Hong JS (2005). Microglia and inflammation-mediated neurodegeneration: multiple triggers with a common mechanism. *Progress in Neurobiology*.

[32] Ransohoff RM, Perry VH (2009). Microglial physiology: unique stimuli, specialized responses. *Annual Review of Immunology*.

[33] Zang L-Y, Misra HP (1993). Generation of reactive oxygen species during the monoamine oxidase-catalyzed oxidation of the neurotoxicant, 1-methyl-4-phenyl-1,2,3,6- tetrahydropyridine. *Journal of Biological Chemistry*.

[34] Muralikrishnan D, Mohanakumar KP (1998). Neuroprotection by bromocriptine against 1-methyl-4-phenyl-1,2,3,6- tetrahydropyridine-induced neurotoxicity in mice. *The FASEB Journal*.

[35] Oyagi A, Oida Y, Hara H (2008). Protective effects of SUN N8075, a novel agent with antioxidant properties, in *in vitro* and *in vivo* models of Parkinson’s disease. *Brain Research*.

[36] Zhang J, Perry G, Smith MA (1999). Parkinson's disease is associated with oxidative damage to cytoplasmic DNA and RNA in substantia nigra neurons. *The American Journal of Pathology*.

[37] Kasture S, Mohan M, Kasture V (2013). *Mucuna pruriens* seeds in treatment of Parkinson's disease: pharmacological review. *Oriental Pharmacy and Experimental Medicine*.

[38] Beal MF (2002). Oxidatively modified proteins in aging and disease. *Free Radical Biology and Medicine*.

[39] Fahn S, Cohen G (1992). The oxidant stress hypothesis in Parkinson's disease: evidence supporting it. *Annals of Neurology*.

[40] Miller RL, James-Kracke M, Sun GY, Sun AY (2009). Oxidative and inflammatory pathways in parkinson's disease. *Neurochemical Research*.

[41] Kadota T, Yamaai T, Saito Y (1996). Expression of dopamine transporter at the tips of growing neurites of PC12 cells. *Journal of Histochemistry and Cytochemistry*.

[42] Dauer W, Przedborski S (2003). Parkinson's disease: mechanisms and models. *Neuron*.

[43] Zhang ZT, Cao X, Xiong N (2010). Morin exerts neuroprotective actions in Parkinson disease models *in vitro* and in *vivo*. *Acta Pharmacologica Sinica*.

[44] Vitvitsky V, Thomas M, Ghorpade A, Gendelman HE, Banerjee R (2006). A functional transsulfuration pathway in the brain links to glutathione homeostasis. *The Journal of Biological Chemistry*.

[45] Lin M, Yu Y, Chen K (2011). Kaempferol from Semen cuscutae attenuates the immune function of dendritic cells. *Immunobiology*.

